# Low-intensity extracorporeal shockwave therapy for erectile dysfunction

**DOI:** 10.1080/2090598X.2021.1948158

**Published:** 2021-07-05

**Authors:** Onder Canguven, Kareim Khalafalla, Abdulla Al Ansari

**Affiliations:** Urology Department, Hamad General Hospital, Doha, Qatar

**Keywords:** Low-intensity extracorporeal shockwave therapy, erectile dysfunction, erection, male

## Abstract

**Objectives**: To review the current evidence of clinical effectiveness of low-intensity extracorporeal shockwave therapy (LI-ESWT) treatment for erectile dysfunction (ED).

**Methods:**

A selective database search using Medical Subject Headings (MeSH) terms ‘low intensity extracorporeal shock wave therapy’ and ‘erectile dysfunction’ was conducted in accordance with the Preferred Reporting Items for Systemic Reviews and Meta Analyses (PRISMA) guidelines to review the effectiveness of LI-ESWT for ED. We performed a systematic search of publications using the PubMed and Web of Science databases (January 2010–December 2020) for prospective randomised clinical trials (RCTs). The success rate of LI-ESWT associated with ED were recorded and analysed.

**Results:**

A total of 106 articles were reviewed after searching for the keywords. Overall, 11 RCTs were included in this systematic review. A total of 920 male patients were treated in 11 RCTs. The patients’ ages ranged from 18 to 80 years and they had ≥3 months of ED symptoms. Vasculogenic and neurogenic causes were addressed in 81% and 19% of patients, respectively. Of the 920 patients, 348 patients had a statistically significant improvement in their erectile function after LI-ESWT; however, 572 did not have a statistically significant improvement.

**Conclusions:**

The present review found that LI-ESWT has a role in ED treatment in laboratory studies, but its role in human clinical trials is still controversial. Further good quality studies need to be conducted to properly assess its true potential in ED treatment.

## Introduction

Erectile dysfunction (ED) is an increasing problem for men and it affects both their quality of life and that of their loved ones. Vasculogenic ED is due to diseases such as diabetes mellitus, hypertension, hyperlipidaemia, smoking, or vascular occlusive disease [1]. The United States Food and Drug Administration (FDA)-approved oral phosphodiesterase type 5 inhibitors (PDE5is) available for management of ED in the USA include sildenafil, tadalafil, vardenafil, and avanafil. Several other PDE5is have been approved for use in other countries [2]. Men with ED who are not satisfied with PDE5i therapy should be informed regarding the treatment option of a vacuum erection device, intraurethral alprostadil and intracavernosal injections, including discussion of benefits and risks/burdens [2].

Advances in ED management can be expected to continue into the future in parallel with ongoing progress in the field of sexual medicine more broadly. Developments in healthcare delivery, diagnostics, and therapeutics will be the underpinnings of improved, evidence-based clinical practice in this field. Scientific discovery in the vascular biology and neurophysiology of penile erection will continue to take centre stage, with particular focus on molecular and cellular signalling pathways and growth factor mechanisms that may be exploited to produce the next generation of pharmacotherapeutics, as well as gene, stem cell, and regenerative therapies.

Low-intensity extracorporeal shockwave therapy (LI-ESWT) is a ‘hot topic’ in the field of ED, both in the medical community and the common media, due to widespread advertising of this treatment. From the physical point of view, a shockwave is defined by an abrupt, nearly discontinuous change in pressure and by having a velocity that is higher than the speed of sound in the medium it propagates. Alarmingly, this treatment is being offered without what many authorities believe is adequate data. The ideal patient population for LI-ESWT and defining important technical parameters (number of shocks, energy level, location of probe application, number/timing of sessions) have yet to be fully defined [3]. Moreover, the term ‘shockwave therapy’ is used loosely, and not all of the currently used machines actually generate focussed shockwaves. Terms such as ‘radial waves’, ‘acoustic waves’, ‘sound waves’, ‘radial shockwaves’, or ‘radial pulse therapy’, while synonymous with each other, are sometimes used interchangeably with shockwaves, despite, based on physics, being a different technology. It is believed that these acoustic waves carry energy, and when targeted and focussed, interact with the targeted deep tissues causing mechanical stress and micro trauma; hence, its effect on erectile tissue can be explained.

Multiple LI-ESWT machines used for ED trials have different transducers with unique technology [4–6]. The consensus statement from the International Society for Medical Shockwave Treatment states that research studies need to specify all of the following factors: device settings, machine used, transducer used, intensity levels applied, coupling medium, and depth of penetration of the device [7].

To further clarify the role of LI-ESWT on ED, we aimed to identify and review the randomised clinical trials (RCTs) that have been performed to evaluate the effectiveness of LI-ESWT for ED.

## Methods

### Search strategy

A systematic literature search was conducted in accordance with the Preferred Reporting Items for Systemic Reviews and Meta Analyses (PRISMA) guidelines using the PubMed and Web of Science databases, on 1 January 2021. We retrieved all published articles between January 2010–December 2020 using the Medical Subject Headings (MeSH) terms for a ‘low intensity extracorporeal shock wave therapy’ and ‘erectile dysfunction’.

### Study selection

The generated list of articles was screened by title and abstract by the two authors (K.K. and O.C.) and then relevant full papers were examined. Review articles were also explored to find additional appropriate papers. Data were then extracted cross-checked and verified. For the specific purpose of this study, inclusion criteria were human studies performed in men and published in English. Studies were considered if they were RCTs investigating the effect of LI- ESWT for the treatment of ED. Exclusion criteria were female gender, animal species, non-English language and other study types, i.e. case report, case series, review articles and meta-analysis.

**Figure 1.**
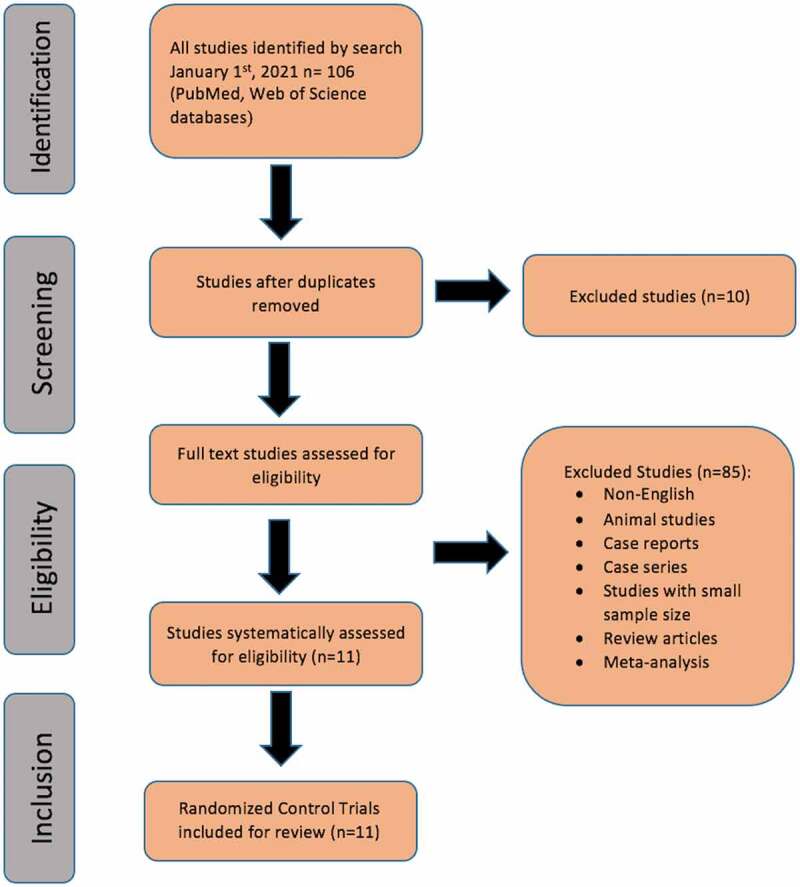
Study flowchart

Results of RCTs were considered to provide clinical statements on the use of LI-ESWT for the treatment of ED (Figure 1).

### Outcome measures

The outcomes of interest were as follows: aetiology of ED, methods of assessment for improvement of erection, number of shockwave sessions/week, total impulses, frequency and number of shockwaves/min, follow-up time period, and mean results for the International Index of Erectile Function-Erectile Function domain (IIEF-EF), Erection Hardness Score (EHS), and Sexual Encounter Profile question 3 (SEP3) questionnaires. Relevant results are tabulated in Table 1 [8–18].

**Table 1.** Summary of RCTs included

Reference	Aetiology of ED	Study population, *n*	Methodology	Session/week (total *n*)	Impulses (*n*)/pulses (*n*/min)/frequency (Hz)	Follow-up, weeks	Pre-/post-treatment IIEF-EF (score); PDU (PSV, cm/s); EHS (score); SEP3 (yes/no); CGIC
Vardi *et al*. [14]	Vasculogenic	67	IIEF-EF EHS Plethysmography	2/week (12)	300/120/NA	12	IIEF-EF (12.6/19.3)* Plethysmography*
Olsen *et al*. [12]	Vasculogenic	105	IIEF-EF EHS	1/week (5)	3000/NA/5	5	IIEF-EF 32% and 15% increase
Yee *et al*. [15]	Vasculogenic	58	IIEF-EF EHS	2/week (12)	1500/120/NA	13	IIEF-EF (17.8/15.8) EHS (2.7/2.4)
Srini *et al*. [13]	Vasculogenic	135	IIEF-EF EHS CGIC	2/week (12)	300/120/NA	48	IIEF-EF (9.5/18.2)* EHS* CGIC*
Kitrey *et al*. [17]	Vasculogenic	58	IIEF-EF EHS CGIC	2/week (12)	1500/120/NA	4	Median change IIEF-EF: (7/13)*
Kalyvianakis *et al*. [9]	Vasculogenic	46	IIEF-EF PDU	2/week (12)	1500/160/NA	48	IIEF-EF (13.8/19.1)* PDU (31.3/35.5)*
Fojecki *et al*. [18]	Vasculogenic	126	IIEF-EF EHS	1/week (10)	600/NA/5	48	IIEF-EF (11.2/14.3)* and (10.9/12.8)
Kalyvianakis *et al*. [10]	Vasculogenic	42	IIEF-EF SEP PDU	1/week (12) and 2/week(18)	5000/NA/8	24	IIEF-EF (15.8/19.9)* SEP3 (38.1%56.3%)* PDU, PSV change (mean [SD] +4.9 [2.5] cm/s)* and (20.3/22.1)*
Katz *et al*. [11]	Vasculogenic	78	IIEF EHS	1/day (5) and 1/every other day (6)	3600/NA/NA	24	IIEF-EF (18.3/NA) and (17.6/21.8)* EHS (2.6/NA) and EHS (2.7/NA)
Zewin *et al*. [16]	Neurogenic: post-cystoprostatectomy	128	IIEF EHS PDU	2/week (12)	300/120/NA	36	IIEF-EF (27.9/24.2) PDU (46.5/36.5)
Baccaglini *et al*. [8]	Neurogenic: post-prostatectomy	77	IIEF-5	1/week (8)	2400/300/5	16	IIEF-5 (10.3/12.7)*

CGIC: Clinical Global Impression of Change; NA: not available. *P < 0.01.

## Results

Using the keywords ‘Low intensity extracorporeal shock wave therapy’ and ‘erectile dysfunction’ led to the identification of 106 publications. These 106 publications included reviews, clinical trials and case reports. When we limited our research to RCTs, 11 publications were identified [8–19].

The 11 RCTs included patients aged 18–80 years. The patients reported having a stable heterosexual relationship for >3–6 months and presented with a 3–6-month history of ED. All patients were subjected to a 2–4-week washout period from previous PDE5i usage. The cause of ED was vasculogenic in nine of the 11 studies and in the remaining two, one was post-radical prostatectomy and the other post-cystoprostatectomy (Table 1).

All RCTs in the present review used the IIEF-EF and EHS questionnaires as a baseline assessment for the erectile function of the included patients in the trials.

Three studies assessed penile haemodynamics as an objective method via the peak systolic velocity (PSV) and resistive index by penile Doppler ultrasonography (PDU) [9,10,16], while one study used veno-occlusive strain gauge plethysmography [14], in which penile blood flow is measured at rest and after a 5-min ischaemic period.

The LI-ESWT protocol was different in each trial, mostly 1–2 sessions/week were administered to these patients with differences in randomisation of the groups, cross-over treatment sessions between placebo sham/control, and active treatment groups. The mean (range) number of sessions was 12 (5–18). The settings of the devices used ranged from 300 to 5000 pulses, with an average energy of 0.09 mJ/mm^2^ (120–300 pulses/min and a frequency of 5–8 Hz) in the sessions provided. The follow-up period ranged from 1 to 12 months after the completion of the LI-ESWT protocol for ED treatment.

Of the 11 RCTs reviewed in the present study, five studies reported a statistically significant improvement in questionnaire scores. Four of the 11 studies reported a statistically nonsignificant improvement according to the questionnaires administered before and after LI-ESWT in patients with ED. Two of the 11 studies did not show any improvement at all.

## Discussion

The present systematic review of 11 RCTs involving 920 patients demonstrated conflicting results for the effect of Li-ESWT on ED with regards to the IIEF-EF questionnaire improvement and other assessment methods used before and after treatment. A total of five studies reported a statistically significant role of shockwave therapy in ED, while four studies had a positive result but with a *P* > 0.05. Finally two RCTs reported no effect at all for LI-ESWT on ED. All the published RCTs compared the effect of LI-ESWT on patients with ED to another group with sham therapy.

According to the energy level, the ESWT can be divided into high-intensity ESWT and LI-ESWT energy. Although both treatment modalities are therapeutic, the high-intensity ESWT is typically administered for destruction of solid aggregations inside or outside tissues, whereas LI-ESWT treatment is used for tissue repair and regeneration [20]. Multiple studies have reported the benefit of LI-ESWT in different medical aspects such as musculoskeletal disorders, wound and bone healing disturbances, ischaemic heart diseases and spastic hypertonia [21–25]. Recently, LI-ESWT has been successfully used in the field of regenerative medicine after its original introduction as a urological lithotripsy [20].

LI-ESWT has been investigated, both in humans and animals with ED, in multiple studies over the years. Vardi *et al*. [14] published the first randomised, double-blind, sham controlled study on LI-ESWT and ED. Vardi *et al*. [14] showed that LI-ESWT has a positive short-term clinical and physiological effect on erectile function (mean [SD] increase in IIEF-EF domain scores was 11.5 [0.86] and 12.6 [0.75], in the sham and treatment groups, respectively). The major limitation of the Vardi *et al*. [14] study was the short follow-up period of 12 weeks after the treatment sessions.

Kalyvianakis *et al*. [9] assessed the penile haemodynamic changes after LI-ESWT in patients with ED by introducing PDU in a double-blinded sham-controlled trial. They did PDU before the LI-ESWT treatment sessions and during 3 months of follow-up to support the results of the trial. The mean change of PSV was 4.5 and 0.6 cm/s for the treatment and sham-control groups, respectively. They also reported minimal clinically important differences in the IIEF-EF for the active vs sham group of 56.7% vs 12.5% at 1 month, 56.7% vs 12.5% at 3 months, 63.3% vs 18.8% at 6 months, 66.7% vs 31.3% at 9 months, and 75% vs 25% at 12 months [9].

A year later, Kalyvianakis *et al*. [10] conducted a different clinical trial assessing the effect and safety of a difference in the number of sessions, frequency and repetition on erectile function. The latter researchers divided patients into Group A: LI-ESWT therapy session 1/week and Group B: 2/week for 6 consecutive weeks into two phases. Patients were followed for 6 months. Kalyvianakis *et al*. [10] reported an improved effect on erection and sexual performance, with an increase in the total session numbers, frequency of sessions/week and repetition of treatment within 6 months without any further side-effects. Srini *et al*. [13] reported on their 12-month follow-up after LI-ESWT and found significant increases in the IIEF-EF and EHS domains. However, these results are seriously flawed by a very high dropout rate (58% and 42% in sham and active treated groups, respectively).

Neurogenic ED after radical prostatectomy or cystoprostatectomy is believed to be due to injury in the neurovascular bundles. This may occur by partial or total sectioning or by stretching. Baccaglini *et al*. [8] conducted the first trial assessing the LI-ESWT role on ED after prostatectomy. A difference between groups was detected when assessing the final median IIEF-5 score. However, the primary clinical endpoint considering a difference of >4 points between the arms was not reached. After therapy with 19,200 impulses of therapy across 8 weeks, they found a statistically nonsignificant improvement in the IIEF-5 score [8].

Zewin *et al*. [16] also explored the role of LI-ESWT in penile rehabilitation in men who underwent nerve-sparing radical cystectomy. Patients were allocated in three groups: LI-ESWT, PDE5i and control groups. In the three groups, statistical evaluation showed a significant increase in the total IIEF score, orgasm, desire, intercourse satisfaction, and overall satisfaction domains scores, and the EHS throughout the follow-up period [16]. The overall comparisons among the three groups according to PDU over 3, 6, and 9 months of follow-up, showed no significant difference in the end-diastolic velocity [16]. Also, PSV did not exhibit any significant changes over time among the study groups [16].

The present data suggest a variable effect of LI-ESWT on erectile function up to 12 months after treatment. However, according to guidelines, the clinical long-term significance of this improvement is uncertain (level 2; grade C) [2]. This recommendation was also confirmed in our present review. The present review assessed the effects of LI-ESWT on ED in 11 RCTs performed up to January 2021. The results indicated that majority of the 11 RCTs (six) showed insignificant improvements in erectile function. It appears that patients with ED due to radical pelvic surgeries have very limited chance of recovery of erectile function and minimal benefit from LI-ESWT; moreover, more data are needed to assess the longer-term effects of LI-ESWT.

## Conclusion

There could be several reasons for the conflicting results on the effect of LI-ESWT in the literature. Current evidence is promising but is still controversial; therefore, a clear clinical recommendation of LI-ESWT for ED cannot be made, and more high-quality studies are needed. Patients should be informed about the conflicting results regarding the effectiveness of this treatment when discussing LI-ESWT.

**Table:** 

Study Name	Etiology of ED	Study Population	Methodology	Session/Week (Total n)	Impulse (number) Pulse (number/min) Frequency (Hz)	Follow up (weeks)	Pre-/Post-treatment IIEF-EF (score); PDU (PSV: cm/sec); EHS (score); SEP3 (Yes/No); CGIC
Vardi et al [14]	Vasculogenic	67	IIEF-EF EHS Plethysmography	2/Week (n = 12)	300/120/NA	12	IIEF-EF (12.6/19.3)* Plethysmography*
Olsen et al [12]	Vasculogenic	105	IIEF-EF EHS	1/Week (n = 5)	3,000/NA/5	5	IIEF-EF 32% and 15% increase
Yee et al [15]	Vasculogenic	58	IIEF-EF EHS	2/Week (n = 12)	1,500/120/NA	13	IIEF-EF (17.8/15.8) EHS (2.7/2.4)
Srini et al [13]	Vasculogenic	135	IIEF-EF EHS CGIC	2/Week n = 12	300/120/NA	48	IIEF-EF (9.5/18.2)* EHS* CGIC*
Kitrey et al [17]	Vasculogenic	58	IIEF-EF EHS CGIC	2/Week (n = 12)	1,500/120/NA	4	Median change IIEF-EF: (7/13)*
Kalyvianakis et al [9]	Vasculogenic	46	IIEF-EF PDU	2/Week (n = 12)	1,500/160/NA	48	IIEF-EF (13.8/19.1)* PDU (31.3/35.5)*
Fojecki et [18]	Vasculogenic	126	IIEF-EF EHS	1/Week (n = 10)	600/NA/5	48	IIEF-EF (11.2/14.3)* and (10.9/12.8)
Kalyvianakis et al [10]	Vasculogenic	42	IIEF-EF SEP PDU	1/week (n = 12) and 2/Week(n = 18)	5,000/NA/8	24	IIEF-EF (15.8/19.9)* SEP3 (38.1%56.3%)* PDU [PSV change (+4.9 ± 2.5 cm/s)* and (20.3/22.1)*]
Katz et al [11]	Vasculogenic	78	IIEF EHS	1/Day (n = 5) and 1/Every other day (n = 6)	3600/NA/NA	24	IIEF-EF (18.3/NA) and (17.6/21.8)* EHS (2.6/NA) and EHS (2.7/NA)
Zewin et al [16]	Neurogenic: Post-Cystoprostatectomy	128	IIEF EHS PDU	2 / Week (n = 12)	300/120/NA	36	IIEF-EF (27.9/24.2) PDU (46.5/36.5)
Baccaglini et al [8]	Neurogenic: Post-Prostatectomy	77	IIEF-5	1/Week (n = 8)	2400/300/5	16	IIEF-5 (10.3/12.7)*

## Abbreviations

ED: erectile dysfunction; EHS: Erection Hardness Score; IIEF-5: International Index of Erectile Function-5; IIEF-EF: IIEF-Erectile Function domain; (LI-)ESWT: (low-intensity) extracorporeal shockwave therapy; PDE5i: phosphodiesterase type 5 inhibitor; PDU: penile Doppler ultrasonography; PSV: peak systolic velocity; RCT: randomised control trial; SEP3: Sexual Encounter Profile question 3
